# Mutations in the quinolone resistance determining region in *Staphylococcus epidermidis* recovered from conjunctiva and their association with susceptibility to various fluoroquinolones

**DOI:** 10.1136/bjo.2007.129858

**Published:** 2008-06

**Authors:** M Yamada, J Yoshida, S Hatou, T Yoshida, Y Minagawa

**Affiliations:** 1 Division for Vision Research, National Institute of Sensory Organs, National Tokyo Medical Center, Tokyo, Japan

## Abstract

**Background:**

*Staphylococcus epidermidis* is one of the prominent pathogens in ocular infection. The prevalence of mutations in the quinolone resistance determining region (QRDR) area in *S epidermidis* isolated from the ocular surface and its association with fluoroquinolone resistance has not been fully elucidated.

**Methods:**

Mutations in the QRDR of *gyrA*, *gyrB*, *parC*, and *parE* genes of 138 isolates of *S epidermidis* recovered from the human conjunctival flora were analysed. The minimal inhibitory concentrations (MICs) of four fluoroquinolones (levofloxacin, gatifloxacin, moxifloxacin and tosufloxacin) against these isolates were also determined using agar dilution methods.

**Results:**

The MIC_90_ values of levofloxacin, gatifloxacin, moxifloxacin and tosufloxacin were 3.13, 1.56, 0.78 and 3.13 μg/ml, respectively. The MIC values of all fluoroquinolones showed a bimodal distribution (susceptible strain and less susceptible strain). Mutations with amino acid substitution in the QRDR were present in 70 (50.7%) isolates. 19 different combinations of mutations were detected: 3 isolates (2.2%) had four mutations, 8 (5.8%) had three mutations, 43 (31.2%) had double mutations and 16 (11.6%) had single mutations. Isolates with mutations in the QRDR of both *gyrA* and *parC* (n = 53) were less susceptible to fluoroquinolones.

**Conclusions:**

The present findings show that approximately half the *S epidermidis* isolates from the normal human conjunctiva have mutation(s) in the QRDR. The presence of mutations in both *gyrA* and *parC* is strongly associated with reduced susceptibility to fluoroquinolones.

*Staphylococcus epidermidis* is one of the most prominent causes of conjunctivitis, keratitis and endophthalmitis.^1^^–^8 Although the relative frequency of different organisms as causative agents in keratitis varies during different periods and in different geographical regions, *S epidermidis* is among the most frequently encountered organisms in clinical studies conducted in the USA, Germany and Japan.^3^^–^5 It is the most common bacterial isolate in most large studies of acute postoperative endophthalmitis.^7^ ^8^

The fluoroquinolones are the newest family of antibacterial agents used in the treatment of ocular infections.^2^ ^3^ ^9^^–^11 In Japan, ofloxacin was the first fluoroquinolone introduced for topical ophthalmic use in 1987. Since then, six other fluoroquinolones—norfloxacin, lomefloxacin, levofloxacin (LVFX), gatifloxacin (GFLX), tosufloxacin (TFLX) and moxifloxacin (MFLX)—have been approved for clinical use as eye drops in Japan. In addition to these compounds, ciprofloxacin has been used clinically in other countries. Their bactericidal activity against the most frequently observed Gram-positive and Gram-negative ocular pathogens is generally excellent and their high potency has made them a common choice for the treatment and prevention of ocular infections.

However, as with other antibiotic agents, continued use in a population raises the issue of emerging resistance.^12^^–^14 Since the introduction of fluoroquinolones for ophthalmic use, the reported incidence of in vitro resistance to fluoroquinolones in bacteria isolated from cases with bacterial keratitis and endophthalmitis has been steadily increasing. A previous study reviewed the database of bacterial flora cultured from the conjunctival sac of 1455 Japanese patients scheduled for intraocular surgeries between 1995 and 2002.^14^ The incidence of in vitro resistance of bacterial isolates to ofloxacin increased from 13.5% in 1995 to 32.8% in 1999. Moreover, when ofloxacin was replaced by LVFX in 2000, the incidence of resistance to LVFX gradually increased from 14.5% in 2000 to 20.5% in 2002.

The primary targets of fluoroquinolones are two essential enzymes of bacterial cells, DNA gyrase and topoisomerase IV.^15^^–^17 In *S epidermidis*, DNA gyrase is composed of the GyrA and GyrB subunits encoded by the *gyrA* and *gyrB* genes, respectively. Topoisomerase IV is composed of ParC and ParE subunits encoded by *parC* and *parE* genes, respectively. In most bacterial species, mutations occur in the highly conserved quinolone resistance-determining regions (QRDR) of the genes that encode DNA gyrase and topoisomerase IV. In *Staphylococcus aureus*, several studies have shown that a combination of mutations in both genes can cause high-level resistance even to the newer fluoroquinolones.^18^^–^21 However, the prevalence of mutations in the QRDR in *S epidermidis* isolated from the ocular surface and its association with fluoroquinolone resistance have not been fully investigated.^15^^–^17 The present study analysed mutations in the QRDR of *gyrA*, *gyrB*, *parC* and *parE* genes of 138 isolates of *S epidermidis* recovered from conjunctival flora. The susceptibility of these isolates to LVFX, GFLX, MFLX and TFLX was also determined.

## METHODS

### Bacterial isolates and susceptibility testing

One hundred and thirty-eight isolates of *S epidermidis* were collected from the conjunctival sac of 138 eyes of 129 patients who were scheduled for intraocular surgery at the National Tokyo Medical Center between November 2004 and June 2005. The mean (SD) age of the patients was 70.7 (14.9) years (range 6–91 years). The patients had not received either ophthalmic or systemic antibiotics prior to bacterial sampling.

Scrapes of the inferior conjunctival fornix were taken in the absence of topical anaesthetic using a sterile cotton swab. The samples were immediately inoculated into Mueller-Hinton (MH) agar and incubated at 35°C in air for 16–20 h for the selection of staphylococci. The MicroScan WalkAway-96 (Baxter Japan, Tokyo) with MicroScan Rapid Pos Combo Panel (Baxter) was used for the identification of *S epidermidis*. Positive cultures were stored at −80°C until the agar dilution testing to determine the minimum inhibitory concentration (MIC).

MICs for LVFX, GFLX, MFLX and TFLX were determined by the agar dilution method in accordance with the recommendations of the Japanese Society of Chemotherapy.^22^ The bacterial suspensions in saline were inoculated on MH agar plates supplemented with defined concentrations of drugs. The plates were incubated at 35°C under aerobic conditions and MICs were determined after 20–24 h of incubation. Drug concentrations ranged from 0.025 μg/ml to 100 μg/ml in twofold increments except for TFLX (0.025 μg/ml to 25 μg/ml) because of its limited solubility.

### DNA amplification and sequencing of QRDR

The isolates were suspended in tryptic soy broth and cultured overnight. Genomic DNA was extracted using the Wizard SV 96 genomic DNA purification system (Promega KK, Japan). One μl of the genomic DNA solution was applied in 20 μl of amplification mixture (5 pM each primer, 1.6 μl dNTP mixture, 2 μl Ex Taq buffer and 0.1 μl LA Taq (Takara Bio Inc, Japan)). Polymerase chain reaction (PCR) amplification was performed with the primers as shown in table 1. PCR primers were selected from the published sequences of *S epidermidis* RP62A. Each reaction was amplified with the following temperature profiles: 30 cycles at 94°C for 30 s, 55°C for 30 s and 72°C for 1 min. The amplified DNA products were separated and identified by 2% agarose gel electrophoresis.

**Table 1:** Primers used in the study. Nucleotide positions are indicated according to GenBank sequence number NC 002976 (*S epidermidis* RP62A)

Target gene	Primer sequence (5′ to 3′)	Product size(bp)	Position
*gyrA*	ATGCGTGAATCATTCTTAGACTATGC	284	2 609 699–2 609 724
	GAGCCAAAGTTACCTTGACC		2 609 441–2 609 460
*gyrB*	CAGCATTAGACGTTTCAAG	251	2 610 508–2 610 528
	CCAATACCCGTACCAAATGC		2 610 278–2 610 297
*parC*	TCGCAATGTATTCAAGTGGG	197	939 185–939 204
	ATCGTTATCGATACTACCATT		939 361–939 381
*parE*	AAGCTCAACAAGCACGCGAGGCTG	324	938 196–938 219
	TTAAAGTCAGTACCAACACCAGCAC		938 493–938 520

PCR products were purified using ExoSAP according to the manufacturer’s instructions (GE Healthcare Bio-Sciences KK, Japan). PCR-amplified DNA was sequenced by the dye terminator method in both the forward and reverse directions. Using Phred/Phrap/Polyphred software, the quality score of each base was calculated. Sample sequences were compared with a reference sequence and mutations were detected. The strain *S epidermidis* ATCC 35984 (RP62A) was used as a reference.

## RESULTS

The mutations identified in the QRDR of the *gyrA*, *gyrB*, *parC* and *parE* genes are summarised in table 2. Nineteen different combinations of mutations were identified in 70 isolates, whereas no mutations were detected in 68 isolates. Three isolates (mutation profile type 2, 9 and 12) had four amino acid substitutions, 8 isolates (mutation profile type 4, 5 and 8) had three amino acid substitutions, 43 isolates (mutation profile type 1, 3, 6, 7, 10, 11 and 13) had double amino acid substitutions and 16 isolates (mutation profile type 14–19) had single amino acid substitutions.

**Table 2:** Mutations in the quinolone resistance determining regions of *gyrA*, *parC* and *parE* in 70 strains *of Staphylococcus epidermidis*

Mutation type	No of isolates	Mutation			
		*gyrA*	*gyrB*	*parC*	*parE*
1	28	Ser84Phe	–	Ser80Tyr	–
2	1	Ser84Phe	–	Ser80Tyr + Asp84Val + Ala85Ser	–
3	4	Ser84Phe	–	Ser80Phe	–
4	4	Ser84Phe	–	Ser80Phe + Asp84Tyr	–
5	1	Ser84Phe	–	Ser80Phe + Asp84Asn	–
6	2	Ser84Phe	–	Asp84Tyr	–
7	5	Ser84Tyr	–	Ser80Phe	–
8	3	Ser84Tyr	–	Ser80Phe	Asp434Asn
9	1	Ser84Tyr + Glu88Lys	–	Ser80Phe + Asp84Ala	–
10	2	Ser84Tyr	–	Ser80Tyr	–
11	1	Ser84Tyr	–	Ser80Ile	–
12	1	Ser84Ile	–	Ser80Phe	Asn404Ser + Asp434Asn
13	1	–	–	Ser80Tyr	Asn404Ser
14	1	–	–	Ser80Phe	–
15	1	–	–	Asp69Asn	–
16	1	–	–	Ser81Pro	–
17	1	–	–	Asp84Gly	–
18	11	–	–	–	Asn404Ser
19	1	–	–	–	Asp434Asn

In the *gyrA* gene, a single-point mutation was found in 53 isolates at codon 84. Double-point mutations in the *gyrA* gene were identified in 1 isolate at codons 84 and 88 (mutation profile type 9). No mutations were found in the QRDR area of the *gyrB* gene. In the *parC* gene, single-point mutations were found in 51 isolates at codons 69, 80, 81, 84 or 85. Double-point mutations were identified in 6 isolates at codons 80 and 84 (mutation profile type 4, 5 and 9). Triple-point mutations were identified in 1 isolate at codons 80, 84 and 85 (mutation profile type 2). In the *parE* gene, single-point mutations were found in 16 isolates at codon 404 or 434. Double-point mutations were identified in 1 isolate at codons 404 and 434.

The MICs of the four tested fluoroquinolones against *S epidermidis* are shown in table 3. All four fluoroquinolones had a bimodal distribution in all isolates (n = 138). Isolates with no mutations in the QRDR (wild type; n = 68) were susceptible to fluoroquinolones. The modes (the number that appears the most) were 0.2 μg/ml for LVFX, 0.1 μg/ml for GFLX, 0.05 μg/ml for MFLX, and 0.05 μg/ml for TFLX. Isolates with mutations restricted in the QRDR of *parC* and/or *parE* (n = 17) showed similar susceptibilities to fluoroquinolones as wild type strains except for one strain with mutation profile type 18. The modes were 0.2 μg/ml for LVFX, 0.1 μg/ml for GFLX, 0.05 μg/ml for MFLX and 0.05 μg/ml for TFLX. Isolates with mutations in the QRDR of both *gyrA* and *parC* (n = 53) were less susceptible to fluoroquinolones. The modes were 1.56 μg/ml for LVFX, 0.78 μg/ml for GFLX, 0.39 μg/ml for MFLX and 0.78 μg/ml for TFLX. Of these 53 isolates, 51 had amino acid substitutions at GyrA84 and ParC80. One isolate (mutation profile type 9) with two amino acid substations both in GyrA and ParC had the highest MICs (25 μg/ml for LVFX, 12.5 μg/ml for GFLX, MFLX and TFLX, respectively).

**Table 3:** Susceptibility of strains *of S epidermidis* to four fluoroquinolones

	No of isolates with the following MIC (μg/ml)											MIC_50_	MIC_90_
	0.025	0.05	0.1	0.2	0.39	0.78	1.56	3.13	6.25	12.5	25		
All isolates (n = 138)													
LVFX			26	55	4		23	19	7	3	1	0.2	3.13
GFLX		29	52	4	1	24	24	2	1	1		0.1	1.56
MFLX	1	72	9	3	26	16	7	3		1		0.05	0.78
TFLX	35	47	3		2	21	10	10	8	2		0.05	3.13
												Mode	
Wild type (n = 68)													
LVFX			24	44								0.2	
GFLX		26	42									0.1	
MFLX	1	60	7									0.05	
TFLX	30	38										0.05	
												Mode	
Mutations in *parC* and/or *parE* (n = 17)													
LVFX			1	11	4			1				0.2	
GFLX		2	10	4			1					0.1	
MFLX		11	2	3		1						0.05	
TFLX	4	9	3			1						0.05	
												Mode	
Mutations in both *gyrA* and *ParC* (n = 53)													
LVFX			1				23	18	7	3	1	1.56	
GFLX		1			1	24	23	2	1	1		0.78	
MFLX		1			26	15	7	3		1		0.39	
TFLX	1				2	20	10	10	8	2		0.78	

GFLX, gatifloxacin; LVFX, levofloxacin; MFLX, moxifloxacin; TFLX, tosufloxacin.

## DISCUSSION

The primary targets of fluoroquinolones are two essential enzymes of bacterial cells, DNA gyrase and topoisomerase IV.^18^^–^20 In most bacterial species the mutations in the genes that lead to fluoroquinolone resistance are limited to a few point mutations at restricted positions of the genes called QRDR. The present study revealed that approximately half (50.7%) of *S epidermidis* isolates in the human conjunctival flora have mutation(s) in the QRDR area of *gyrA*, *gyrB*, *parC* and *parE* genes.

Fluoroquinolone resistance has been studied intensively in *S aureus*.^18^^–^21 The genes encoding topoisomerase IV in *S aureus* are called *grlA* and *grlB*, which are analogous to *parC* and *parE* in *S epidermidis*, respectively. Fluoroquinolone resistance in *S aureus* is generally associated with two single-point mutations in *gyrA* at codon 84, and in *grlA* at codon 80 or 84. *S aureus* isolates with higher levels of resistance are associated with the second mutation in *grlA* at codon 80 or 84, depending on the position of the first mutation. When the second mutation in *gyrA* occurs at codon 85 or 88, in addition to the first mutation at codon 84, the strain shows the highest fluoroquinolone resistance even to newer fluoroquinolones.^21^

The present QRDR sequencing results indicate that the major mechanism of fluoroquinolone resistance in *S epidermidis* is analogous to that of *S aureus*. Isolates with mutations restricted to the QRDR of *parC* and/or *parE* (n = 17) in this study were similarly susceptible to fluoroquinolones as wild type strains. However, the presence of two mutations (n = 53) in both *gyrA* gene (located at codon 84) and *parC* gene (located at codon 80) have been found to be associated with the development of fluoroquinolone resistance.^15^ ^16^

In this study only one isolate (mutation profile type 9), which was highly resistant to all four fluoroquinolones tested, had two amino acid substitutions both in GyrA and ParC. Previous studies have shown that isolates of *S epidermidis* and *S aureus* with two amino acid substitutions both in GyrA and ParC (GrlA in *S aureus*) have the highest fluoroquinolone resistance. The isolates with this mutation type are reported to be relatively rare in *S epidermidis*^15^ ^16^ and to account for less than 10% in *S aureus*.^18^^–^20 However, a high prevalence (50%) of two amino acid substitutions in both GyrA and GrlA has recently been reported.^21^ The empirical use of newer fluoroquinolones without a proper clinical indication may produce additional resistant strains of *S epidermidis*, as has already occurred with *S aureus*.

One possible limitation of the present study was that the patients were scheduled for intraocular surgery. Bacterial isolates therefore represent conjunctival flora rather than ocular pathogens. However, in common ocular infections such as bacterial conjunctivitis and bacterial keratitis, pathogens are frequently the normal bacterial flora that reside on the ocular surface.^2^^–^6 This is true even in cases of postoperative endophthalmitis, in which *S epidermidis* is the most common bacterial isolate from vitreous aspirates.^7^ ^8^ Organisms isolated from the vitreous were genetically identical to those collected from the ocular surface in 68–82% of patients with postoperative endophthalmitis,^7^ suggesting that the study of in vitro susceptibility to various fluoroquinolones is valid.

Drug resistance is a serious concern in treating ocular infections. The current study showed that approximately half the *S epidermidis* isolates from the conjunctival flora have mutation(s) in the QRDR. Both *gyrA* gene and *parC* gene are associated with the development of fluoroquinolone resistance.
